# Therapeutic hypothermia decreases growth of perihemorrhagic edema and prevents critical increase of intracranial pressure in large intracerebral haemorrhage

**DOI:** 10.1186/cc11272

**Published:** 2012-06-07

**Authors:** Rainer Kollmar, Stefan Schwab, Dimitre Staykov

**Affiliations:** 1 Department of Neurology, Friedrich-Alexander-University of Erlangen-Nuernberg, Erlangen, Germany

## Background

Intracerebral hemorrhage (ICH) accounts up to 15% of all first-ever strokes and is associated with high mortality, morbidity, and disability [1,2]. Main factors contributing to poor outcome within the first days after symptom onset are hematoma size, early hematoma growth, the presence of intraventricular hemorrhage, and the size of perihemorrhagic edema (PHE) [3]. PHE leads to secondary injury by a complex pathophysiological cascade following ICH. Above all, mass effects of PHE can lead to critical increase of intracranial pressure and subsequent herniation. Since the volume of PHE increases within the first days after ICH and is correlated to ICH volume, PHE represents a meaningful target for interventions [3]. Therapeutic hypothermia (TH) is a promising candidate to treat or even prevent PHE. Experimental data indicate that TH is neuroprotective after acute brain injury including ICH and reduces PHE [4]. In a proof of concept study, we investigated the effects of mild TH of 35°C over a period of 10 days in patients suffering from large (>25 ml) ICH and compared these patients with a historical control group [5]. Even with standard treatment, ICH of this size has an extremely high mortality and almost never leads to acceptable neurological outcome [2]. In our study, TH prevented the increase of PHE and led to a superb in-hospital survival rate and an acceptable long-term survival and grade of neurological deficits [5,6]. Because of these promising results, we established an institutional protocol in the Department of Neurology at University Hospital Erlangen and treated patients with large ICH with mild TH. Here, we report data of 20 patients with large ICH treated in our neuro-ICU.

## Materials and methods

All patients with large ICH were treated by a detailed institutional protocol that is in line with our ongoing prospective study [7]. Patients aged over 18 years with primary ICH at the level of the basal ganglia or thalamus and a hematoma volume of over 25 ml on initial CCT were treated by TH. Patients have been treated within the first 12 hours after symptom onset if they had a score on the Glasgow Coma Scale (GCS) of ≤8 at presentation or early worsening by 2 points with subsequent endotracheal intubation and neurointensive care treatment. All patients received invasive ICP measurement by external ventricular drainage or a parenchymal probe. Relatives were informed about the treatment and approved this regimen. Patients have not been treated by TH if any clinical signs of herniation such as pupillomotoric defects or bilateral signs of the pyramidal tract at baseline could be observed or the treating team agreed to a do-not-resuscitate order. Laboratory exclusion criteria included an international normalized ratio >1.5, a thrombocyte count below 70,000/μl or leukocytosis >20,000/μl on admission. The presence of intraventricular hemorrhage has not been an exclusion criterion, since we have a standardized protocol, including external ventricular drainage, intraventricular clot lysis and the use of lumbar drainage. Patients who have been randomized to the control arm of our multicenter randomized controlled trial CINCH [7] are not reported here.

### Intervention of therapeutic hypothermia

Patients were treated with an endovascular catheter-based cooling system (ICY catheter; Zoll Medical, USA) positioned in the femoral vein as described previously [5]. The target temperature has been set to 35.0°C. The body core temperature has been measured by a urinary bladder catheter. As soon as body core temperature drops below 36.0°C, patients are covered by a warming blanket to avoid shivering. Ten days after initiation of TH, patients received slowly, controlled rewarming by 0.1°C/hour. The catheter has been changed at least once during the treatment period, preferably on day 4 ± 0.5 after ICH, or if clinically indicated

### Outcome analysis

Patients have been analysed for in-hospital mortality, mortality and functional outcome (modified Ranking Scale and Barthel Index) on days 90 and 180 after ICH.

### Semiautomatic assessment of ICH and PHE volume

The Siemens Leonardo V software for semiautomatic CT volumetry has been used for assessment of hematoma and perihemorrhagic edema volumes. The procedure has been described in detail before [3].

### Statistical analysis

Statistical tests were performed with SPSS 16.0 software package. Data are given as mean ± standard deviation, if not indicated differently. Normality of distribution was tested using the Shapiro-Wilk and Kolmogorov-Smirnov tests. Absolute edema values were not distributed normally. Accordingly, single comparisons of absolute edema between the two groups at different time points were performed using the nonparametric Mann-Whitney *U *test. All other data were distributed normally. The unpaired *t *test was used for single comparisons of ICH values between the two groups. *P *<0.05 was considered significant.

## Results

### Patient characteristics

A total of 20 patients have been treated with mild hypothermia so far. There have been no significant differences between these patients and our historic control group (*n *= 25; Table 1). Overall, the volume of ICH in the initial CT was large with 57 ± 25 ml for the hypothermia group and 59 ± 31 ml for the control group. The mean volume of PHE after ICH has been calculated from cranial CT. Day 1 indicates the CT before start of hypothermia or standard treatment. Rewarming was started at day 10. A significant difference (*P *<0.05) of the volumes at the specific day between the control and hypothermia group was found [5].

**Table 1:** Patient characteristics (from [5])

	Therapeutic hypothermia (*n *= 20)	Historic control (*n *= 25)
Age (years)	62 ± 9	67 ± 7
GCS	5 (3 to 10)	8 (3 to 10)
ICH volume on admission (ml)	57 ± 25	59 ± 31
PHE on admission (ml)	49 ± 40	40 ± 28
Number of patients with intraventricular hemorrhage	13	14

ICH, intracerebral hemorrhage; PHE, perihemorrhagic edema.

### Volume of PHE assessed in CT

While there was no significant difference between the PHE on days 1 and 2, we found significantly higher volumes of PHE in the historic control group on days 3, 6, 11, and 14. Importantly, PHE did not increase after rewarming at days 11 and 14.

### Complications of therapeutic hypothermia

ICP crisis: none of the patients in the hypothermia group had ICP crisis, in contrast to 11 (44%) patients in the control group.

We detected deep venous thrombosis in one patient in the hypothermia group. Shivering appeared in nine (45%) patients and was treated sufficiently by medication including meperidine and, if needed, muscle relaxants. Pneumonia was the most common complication in hypothermia patients. Nineteen (95%) of the patients developed pneumonia, three suffered from ARDS and sepsis. In contrast, pneumonia appeared in 79% of the control patients. Two (10%) patients in the hypothermia group had a ventriculitis during external ventricular drainage. Five (25%) of the hypothermia patients showed a decrease of thrombocytes. However, the thrombocyte count never dropped below 80,000/μl and no complications associated to this decrease could be detected. Four patients (20%) developed bradycardia of below 40 beats per minute, but only one patient received treatment due to bradycardia. One patient (5%) had a pulmonary embolism during hypothermia. However, we could not detect deep venous thrombosis or any other source for PE.

### Clinical outcome

Eighteen patients survived the first 90 days after ICH. Two showed a mRS of 3, seven had a mRS of 4, and eight a mRS of 5 after 90 days. Follow-up assessment after 1a showed that 17 patients survived ICH. Of the surviving patients, 10 patients had a mRS of 3, 12 had a mRS of 4, and two had a mRS of 5.

## Discussion

PHE develops early after ICH, causes an additional mass effect after ICH, and contributes to secondary brain injury [3,4]. This mass effect can be critical especially in large ICH and leads frequently to ICP crisis and brain herniation [3,5,6]. Therefore, PHE is an important target for therapeutic interventions. Animal experiments and clinical data show that TH is a promising candidate for edema and ICP control [4-6]. We could show in a previously published case series that TH decreased PHE and led to acceptable short-term and long-term survival [5,6]. Therefore, we implicated a TH as the standard care for large ICH in our institution and we initiated a German-Austrian controlled multicenter trial to overcome the shortcomings of our historical control group [7].

Here, we report results of the routine use of prolonged mild therapeutic hypothermia in patients suffering from large ICH at the level of the basal ganglia or thalamus. TH prevented the increase of PHE, prevented ICP crisis and lead to an acceptable long-term outcome compared with the historical control group. Importantly, rewarming did not lead to rebound edema as measured by CT. However, TH was also associated with complications, of which infections were the most frequent ones. As this study underscores the promising results of our proof-of-concept study, we are looking forward to the results of the CINCH study and other clinical data investigating the effects of temperature and temperature management of PHE in large ICH.
